# Constipation

**Published:** 1877-05

**Authors:** 


No person can long remain in good health who is subjected to constipation of the bowels. Constipation is, indeed, one of the most dangerous of all diseases, since more than half the sickness that prevails originates from this cause. We have time and again shown how the blood is poisoned by absorption of fecal matter that is too long retained in the system. To promote a healthy and regular action of the bowels without the use of purgatives—which always result in injury when frequently employed—was considered impossible until the discovery of Nealton's Suppository. We have employed them in our practice for several years, and many of our readers have used them on our recommendation, and we have never heard of an instance where they did not prove entirely satisfactory as a remedy for piles and constipation. We know of many cases of constipation, with impared health, that had existed for years, that have been completely cured by the use of Nealton's Suppository once or twice a day for a month. We are daily in receipt of letters from persons in remote parts of the country, thanking us for having recommended the Nealton Suppository. We like to encourage the use of this most valuable remedy by the many who are suffering for the want of it. We will, therefore, send free to any address a box of Nealton's Suppository on receipt of fifty cents. Address, Hall's Journal of Health, No. 137 Eighth Street, New York.

